# Discordant responses of bone formation and absorption markers in Japanese infants with vitamin D deficiency: a comprehensive matched case–control study

**DOI:** 10.1093/jbmrpl/ziae033

**Published:** 2024-03-18

**Authors:** Keigo Takahashi, Kazushige Ikeda, Kaori Hara-Isono, Akihisa Nitta, Nobuhiko Nagano, Takeshi Arimitsu

**Affiliations:** Division of Neonatology, Department of Pediatrics, Saitama City Hospital, Saitama, 336-8522, Japan; Division of Neonatology, Department of Pediatrics, Saitama City Hospital, Saitama, 336-8522, Japan; Department of Pediatrics, Keio University School of Medicine, Tokyo, 160-8582, Japan; Department of Pediatrics, Saitama Medical Center, Dokkyo Medical University, Saitama, 343-0845, Japan; Department of Pediatrics and Child Health, Nihon University School of Medicine, Tokyo, 173-8610, Japan; Department of Pediatrics, Keio University School of Medicine, Tokyo, 160-8582, Japan

**Keywords:** alkaline phosphatase (ALP), infant, parathyroid hormone (PTH), type I collagen N-telopeptide (NTx), tartrate-resistant acid phosphatase isoform 5b (TRACP-5b), vitamin D deficiency

## Abstract

Vitamin D deficiency during infancy has been associated with increased bone turnover rate and bone mineral loss. However, few studies have examined bone turnover markers (BTMs) for both bone formation and resorption in infants with vitamin D deficiency. Here, we analyzed serum concentrations of 25OHD, intact parathormone (iPTH), and BTMs including total alkaline phosphatase (ALP), tartrate-resistant acid phosphatase isoform 5b (TRACP-5b), and serum type I collagen N-telopeptide (NTx) as well as basic clinical characteristics of 456 infants (626 samples) aged less than 12 mo born at Saitama City Hospital, Japan (latitude 35.9° North) between January 2021 and December 2022. One hundred sixteen infants (147 samples) were classified as having vitamin D deficiency (25OHD < 12.0 ng/mL), and 340 infants (479 samples) had sufficient vitamin D levels (25OHD ≥ 12.0 ng/mL). In addition to 25OHD and ALP, both TRACP-5b and sNTx were measured in 331 infants (418 samples), while 90 infants (105 samples) had only TRACP-5b measured and 101 infants (103 samples) had only sNTx measured. Statistical comparison of 104 subjects each in the vitamin D deficiency and sufficiency groups after matching for the background characteristics revealed that the vitamin D deficiency group had significantly higher levels of ALP and iPTH compared with the sufficiency group (*P* = <.0001, .0012, respectively). However, no significant differences were found in TRACP-5b and NTx levels between the 2 groups (*P* = .19, .08, respectively). Our findings suggest discordant responses between bone formation and resorption markers in subclinical vitamin D deficiency during infancy.

## Graphical Abstract

**Graphical Abstract:**
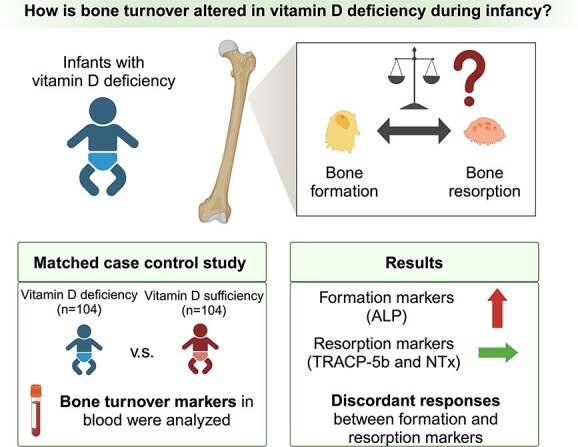

## Introduction

Vitamin D is essential for growth, bone development, and maintenance of optimum bone mass in children and adults.^1^ However, vitamin D deficiency has become a global epidemic. In infants, prolonged vitamin D deficiency in infancy results in disturbed skeletal homeostasis, reduced bone mineralization, slower growth, radiologically evident rickets, and eventually fractures.^2^ Bone growth undergoes intensive modeling during growth, involving both formation and resorption processes. The morphogenesis and remodeling of the bone require a coupled process of bone resorption by osteoclasts followed by bone formation by osteoblasts, which is necessary for normal development and growth.

Determining serum biochemical markers of bone turnover may provide useful indices for evaluating the dynamics of bone metabolism, particularly during infancy when the use of bone densitometry is limited. There are 2 types of bone turnover markers (BTMs)—markers of bone formation and resorption.^2-5^ BTMs of formation include total alkaline phosphatase (ALP), bone alkaline phosphatase (BAP), osteocalcin, and N-terminal propeptide of type I procollagen (P1NP). BTMs of resorption include tartrate-resistant acid phosphatase type 5b (TRACP-5b), type I collagen N-telopeptide (NTx), type I collagen C-telopeptide (CTx), pyridinoline, and hydroxyproline. TRACP-5b is one of the osteoclast enzymes, and its serum concentrations correlate with the number of osteoclasts and bone formation rate. Serum NTx, a direct product of osteoclastic proteolysis, has been used as one of the most responsive and specific indicators of bone catabolism. However, reports on postnatal trends in bone resorption markers are less frequent than those on bone formation markers, including ALP.

Vitamin D deficiency increases bone turnover rate and promotes bone mineral loss. Markers indicating both bone formation and resorption have been documented as elevated in cases of vitamin D deficiency in adolescents, pregnant women, and postmenopausal women.^6-9^ A study on vitamin D-deficient school children showed that supplementation with vitamin D for 6 months was associated with decreasing serum P1NP and CTx levels.^10^ However, few reports have evaluated both bone formation and resorption markers simultaneously in infants with vitamin D deficiency.^11^^,^^12^ Moreover, it is unclear whether testing BTMs beyond ALP is clinically informative in infants with vitamin D deficiency.

This study aimed to examine how bone resorption markers (TRACP-5b, NTx) are altered in comparison to bone formation markers (ALP) in early infants with vitamin D deficiency. Additionally, physiological changes in postnatal bone resorption markers were also evaluated.

## Materials and methods

### Study subjects

This retrospective cohort study was conducted at Saitama City Hospital in Japan which lies 35.9° north between January 2021 and December 2022. Infants born at this hospital who had phlebotomy for any reason up to 12 mo of age in the well-baby nursery or outpatient neonatology clinic were included in the study.^13^^,^^14^ All patients were informed about the study and its opt-out approach in accordance with the guidelines outlined in the “Ethical Guidelines and Policies for Life Sciences and Medical Research Involving Human Subjects” issued by the Ministry of Education, Culture, Sports, Science, and Technology; the Ministry of Health, Labor, and Welfare; and the Ministry of Economy, Trade, and Industry of Japan in 2021.^15^ This study was approved by the Research Ethics Committee of the Saitama City Hospital (H29-31 and R4-72).

Electronic medical records of infants who were tested for 25OHD and ALP, andTRACP-5b and/or NTx were also retrospectively evaluated. Infants born at less than 35 wk of gestation, born to a mother who was treated with medications known to affect calcium or who underwent long-term tocolysis, including magnesium sulfate, due to threatened preterm labor, or born to a mother with any clinical conditions known to affect calcium metabolism were excluded from the study. Demographics, anthropometric measurements, laboratory data, and perinatal history were collected from the electronic medical records. BMI was calculated as weight in kilograms divided by height in meters squared. The 3rd, 50th, and 97th percentiles of BMI values of Japanese infants were according to the Manual for the Assessment of Growth of Japanese Infants and Children in 2012 from the National Center for Child Health and Development.^16^

### Study methods

#### Definition of vitamin D deficiency

The subject infants were classified into 2 groups based on a cutoff value of 12.0 ng/mL for 25OHD.^17^ Perinatal factors, anthropometric measurements, and biochemical measurements including ALP, iPTH, TRACP-5b, NTx, Ca, and IP were compared between the vitamin D deficiency (25OHD < 12.0 ng/mL) and sufficiency (25OHD ≥ 12.0 ng/mL) groups. If the serum 25OHD level was <4.0 ng/mL (lower limit of quantification), then 2.0 ng/mL was used as the value.

#### Statistical handling of multiple testing

In cases where phlebotomy was performed more than once, only the initial test result was used to eliminate arbitrariness.

### The matching method of baseline characteristics

To overcome the bias regarding the different distribution of covariates among patients in both groups, we performed 1:1 matching using a caliper matching method without re-sampling. We matched each case to control with a caliper width of 0.25 using the following 3 variables: age, gestational weeks, and gender.

### Biochemical measurements

In this study, the total serum 25OHD level was assessed via chemiluminescence immunoassay (CLIA) using the Liaison® 25 OH Vitamin D Total Assay with Precision and a Liaison® XL Analyzer (DiaSorin Inc.).^13^^,^^14^ iPTH levels were measured via electrochemiluminescence immunoassay (ECLIA, Elecsys PTH, Roche Diagnostics). TRACP-5b was determined by EIA (Osteolinks ®TRAP-5b; Nittobo Medical Co., Ltd.).^18^ Serum NTx concentrations were evaluated by the OSTEOMARK® NTx serum competitive-inhibition enzyme-linked immunosorbent assay (Abbott Diagnostics Medical Co.).^19^ Serum calcium, magnesium, and inorganic phosphorus levels were measured by a method widely used in Japan.^13^^,^^14^ ALP isozyme was analyzed via Alkaline Phosphatase Isoenzyme kit (QUICK EP, Helena Laboratories).

### Concentration-response relationship with 25OHD

To visually interpret the dose–response correlation between serum 25OHD concentration and 6 variables (ALP, iPTH, TRACP-5b, NTx, Ca, and IP), the restricted cubic spline, a method that can capture a non-linear association, was used for each group. Restricted cubic spline models were used with 3 knots at the cutoff value of 12.0 ng/mL, the 20th, and the 80th percentiles of 25OHD. These percentiles were based on the matched data. The 95% CIs were also calculated.

### Statistical methods

Continuous variables were reported as median (interquartile range [IQR]), while categorized data are reported as *n* (%). Student’s *t*-test or Mann–Whitney test was used to compare means between groups, and Fisher’s exact test was used to compare categorized data. The normality of the distribution was evaluated using the Shapiro–Wilk test. All statistical analyses were 2-tailed, and *P*-values <0.05 were considered statistically significant. A caliper matching was performed using R software (version 4.1.2) with the package “Matching,” and all other statistical analyses were performed using SAS software, version 9.4 (SAS Institute). All figures were generated using Photoshop CS2019 (Adobe). The graphical abstract was created with BioRender.com.

## Results

**Figure 1:**
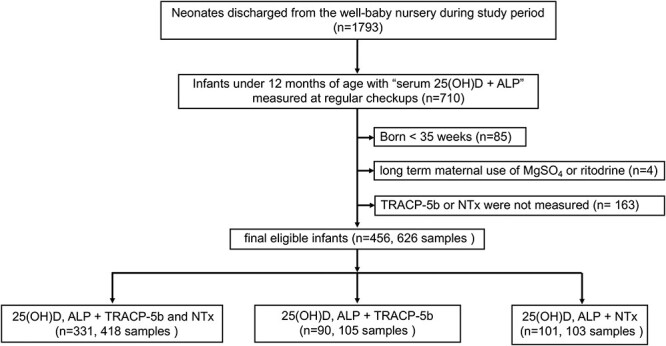
Flowchart describing patient selection.

During the study period, 1793 infants were discharged from the well-baby nursery, of which 456 infants (626 samples) were eligible for analysis. Three hundred thirty-one infants (418 samples) had both TRACP-5b and NTx measured in addition to 25OHD and ALP, while 90 infants (105 samples) had only TRACP-5b measured and 101 infants (103 samples) had only NTx measured (Figure 1).

Baseline characteristics of the study participants (*n* = 456) are summarized in Table 1. Of these, 116 infants (25.4%) were diagnosed with vitamin D deficiency, while 340 infants (74.6%) exhibited sufficient vitamin D levels at the time of phlebotomy. One infant in the vitamin D deficiency group and 5 in the sufficiency group were small-for-gestational age (SGA) (< 3rd percentile) infants (Table 1). One infant in the vitamin D deficiency group and 4 in the sufficiency group had BMIs below the third percentile, whereas 1 in the deficiency group and 6 in the sufficiency group had BMIs over the 97th percentile at data acquisition. Statistically significant differences were observed in weeks of gestation, birth weight, and birth length between the vitamin D deficiency and the sufficiency groups. Considering multicollinearity and known factors affecting BTMs, we performed caliper matching on these 2 groups based on weeks of gestation, age, and sex. After matching, 104 samples were assigned to each group, and the sample size was comparable to a previous report on adults^20^ although maternal age was still significantly different between the 2 groups after matching (Table 1).

**Table 1:** Baseline characteristics before and after matching.

	Before matching (*n* = 456)					After matching (*n* = 208)				
	25OHD < 12.0 ng/mL		25OHD ≥ 12.0 ng/mL			25OHD < 12.0 ng/mL		25OHD ≥ 12.0 ng/mL		
	(*n* = 116)	Missing data	(*n* = 340)	Missing data	*P* value	(*n* = 104)	Missing data	(*n* = 104)	Missing data	*P* value
**Mothers**										
Age	33 [29, 36]	0	33 [30, 37]	0	.15	32 [29, 36]	0	34 [31, 38]	0	.02
Parity	1 [0, 1]	0	1 [0, 1]	0	.18	1 [0, 1]	0	1 [0, 1]	0	.98
Vaginal/Cesarean	62/54	0	202/138	0	.26	57/47	0	50/54	0	.33
**Infants**										
Male/Female	63/53	0	165/175	0	.28	59/45	0	59/45	0	1.00
Gestational weeks	38.3 [37.7, 39.4]	0	38.7 [38.0, 39.9]	0	.02	38.4 [37.9, 39.4]	0	38.4 [37.9, 39.4]	0	.94
Birth weight	2910 [2683, 3118]	0	2999 [2745, 3230]	0	.03	2918 [2723, 3093]	0	2924 [2740, 3176]	0	.52
Birth lengths	48.1 [47.2, 49.5]	0	49.0 [47.7, 50.0]	0	.01	48.1 [47.3, 49.5]	0	48.5 [47.5, 50.0]	0	.21
Days of age	30 [4, 32]	0	30 [4, 33]	0	.28	30 [4, 31]	0	30 [4, 32]	0	.70
SGA (< 3%ile)	1	0	5	0	.62	1	0	3	0	.30
Current weight	3613 [2991, 4186]	0	3794 [3005, 4344]	0	.27	3629 [2970, 4166]	0	3539 [3009, 4161]	0	.92
Current length	51.2 [49.0, 53.0]	1	51.5 [49.5, 53.5]	0	.11	51.1 [48.5, 53.0]	0	50.6 [49.0, 53.0]	0	.80
BMI	13.7 [12.7, 15.0]	1	13.9 [12.4, 15.4]	0	.69	13.8 [12.6, 15.0]	0	13.6 [12.4, 14.9]	0	.54
25OHD (ng/mL)	8.4 [6.8, 11.0]	0	19.5 [16.1, 24.5]	0	<.0001	8.4 [6.8, 11.0]	0	18.7 [15.1, 23.5]	0	<.0001
intact PTH (pg/mL)	36 [27, 51]	43	24 [19, 30]	134	<.0001	38 [27, 55]	41	24 [19, 30]	42	<.0001
ALP (IFCC, U/L)	330 [213, 432]	0	284 [200, 361]	0	.0003	325 [208.5, 431.5]	0	283 [179, 351]	0	.003
ALP isozyme (bone ALP, type 3) (%)	80.4 [79.4, 82.0]	17	80.5 [79.0, 81.5]	147	.35	80.4 [79.4, 82.0]	90	81.0 [79.7, 81.8]	68	.70
TRACP-5b (mU/dL)	2548 [1788, 2964]	22	2519 [1908, 3010]	77	.83	2525 [1752, 2964]	21	2494.5 [1676, 2961]	22	.85
NTx (nM BCE/L)	169.7 [142.4, 193.2]	29	172.0 [148.2, 196.9]	54	.60	170.5 [146.6, 191.0]	26	171.2 [146.1, 190.3]	21	.84
Ca (mg/dL)	10.2 [9.5, 10.5]	0	10.1 [9.7, 10.4]	0	.58	10.1 [9.5, 10.4]	0	10.1 [9.7, 10.4]	0	.58
IP (mg/dL)	6.4 [6.0, 6.8]	0	6.5 [6.1, 6.8]	0	.25	6.4 [6.1, 6.7]	0	6.5 [6.3, 6.8]	0	.03
Mg (mg/dL)	2.1 [2.0, 2.2]	0	2.1 [2.0, 2.2]	0	.59	2.1 [2.0, 2.2]	0	2.1 [2.0, 2.2]	0	.67
Fe (μg/dL)	70.5 [62.5, 83]	56	80 [64, 100]	55	.048	71 [62, 85]	49	75 [63, 100]	25	.54
Zn (mg/dL)	61 [56, 66]	56	62 [56, 70]	75	.32	61 [55, 66]	49	62.5 [56, 69]	30	.50
Crtn (mg/dL)	0.2 [0.2, 0.4]	0	0.3 [0.2, 0.4]	0	.87	0.2 [0.2, 0.4]	0	0.3 [0.2, 0.4]	0	.98
ALB (g/dL)	3.6 [3.4, 3.8]	5	3.6 [3.4, 3.8]	1	.73	3.6 [3.4, 3.8]	3	3.5 [3.4, 3.7]	1	.19
AST (U/L)	29.5 [23, 40]	50	29 [23, 36]	47	.56	29 [23, 40]	45	28 [23, 34]	22	.35
ALT (U/L)	11 [9, 19]	50	13 [9, 19]	47	.65	11 [9, 16]	45	9 [12.5, 19]	22	.38
Blood type		31		97			29		28	
A	26		96			24		24		
B	18		59			14		21		
AB	11		19			10		8		
O	30		69			27		23		

Results are shown as median [Q1;Q3] or numbers.

ALP and iPTH levels were significantly higher in the vitamin D deficiency group compared with those in the vitamin D sufficiency group both before and after matching. After matching, the IP levels in the vitamin D deficiency group were found to be significantly lower than those in the sufficiency group (Table 1). The component of ALP isozyme was also measured in a total of 50 patients after matching, all with type 3 predominance (80.8%, IQR 79.6–81.8). No significant differences in TRACP-5b, NTx, the percentage of type 3 ALP isozyme, and Ca levels were detected between the 2 groups. Serum markers for other organ functions or inflammation known to affect TRACP-5b and NTx levels such as creatinine (Cr), aspartate aminotransferase, and alanine aminotransferase^4^ were within reference ranges in all study subjects (Table 1).

**Figure 2:**
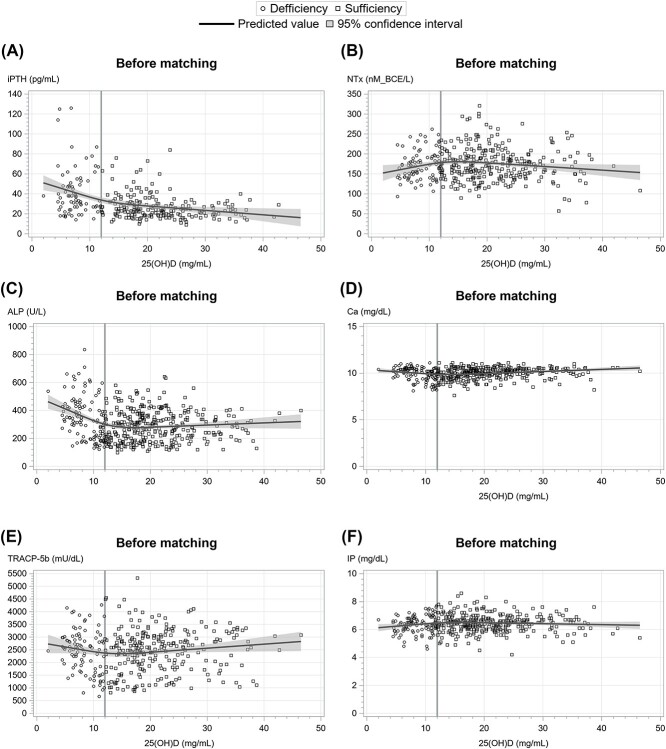
Correlation between serum 25OHD concentrations and (A) iPTH, (B) ALP, (C) TRACP-5b, (D) NTx, (E) Ca, and (F) IP concentrations before matching. Circles and squares represent values from vitamin D deficient and sufficient infants, respectively. The solid lines show cubic regression fit lines.

**Figure 3:**
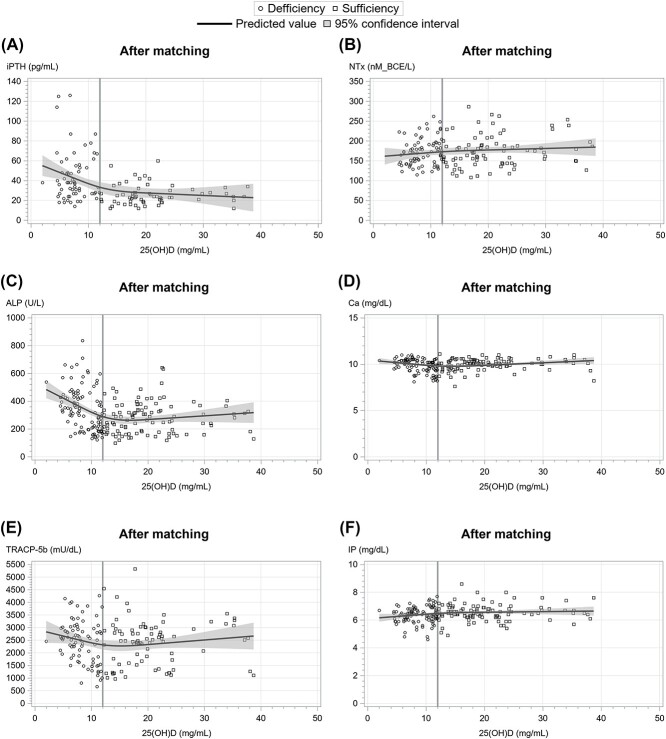
Correlation between serum 25OHD concentrations and (A) iPTH, (B) ALP, (C) TRACP-5b, (D) NTx, (E) Ca, and (F) IP concentrations after matching. Circles and squares represent values from vitamin D deficient and sufficient infants, respectively. The solid lines show cubic regression fit lines.

To investigate dose–response correlations between BTMs and serum 25OHD levels, we selected 6 markers including ALP, iPTH, TRACP-5b, NTx, Ca, and IP as variables and performed cubic spline regression analysis. The spline curves for iPTH and ALP fitted biphasic or negative linear regression, consistent with previous reports (Figures 2 and 3).^13^^,^^21^ In contrast, no characteristic trend was demonstrated for TRACP-5b, NTx, and Ca. (Figures 2 and 3).

**Figure 4:**
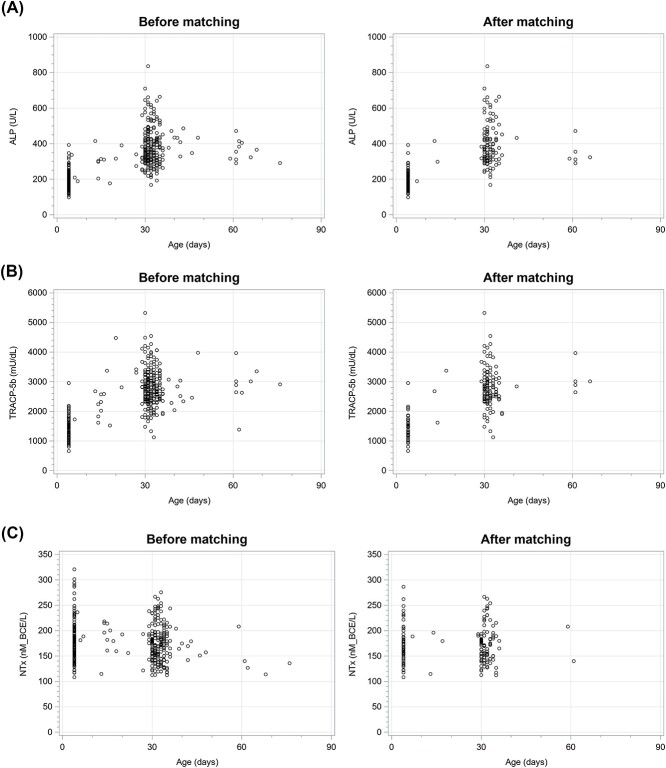
Transition of (A) ALP, (B) TRACP-5b, and (C) NTx up to 90 d of age before and after matching. Only infants whose 25OHD levels were 12.0 ng/mL or higher were included for ALP, whereas all subjects were included for TRACP-5b and NTx.

Lastly, we analyzed the postnatal trend of ALP, TRACP-5b, and NTx up to 180 d in all infants before and after matching (Figure 4). ALP and TRACP-5b were relatively low in the early neonatal period, then increased in the first month of life, and remained high thereafter. Of note, postnatal ALP in this analysis was used only from infants with 25OHD greater than 12.0 ng/mL since ALP is known to be affected by vitamin D status. Our result on the ALP trend is consistent with Zierk’s report.^22^ Conversely, serum NTx was relatively high for 1 mo after birth and gradually decreased thereafter.

## Discussion

In this study, we retrospectively analyzed serum levels of bone resorption markers including TRACP-5b and NTx in combination with bone formation marker ALP in Japanese infants aged less than 12 months with or without vitamin D deficiency. The effects of vitamin D deficiency on BTMs during childhood have been reported only in a few studies,^6^^,^^23^ and to our knowledge, this is the first report of such investigation conducted during early infancy. This study also provides the natural postnatal trend of TRACP-5b and NTx levels in vitamin D-sufficient infants up to 6 months of age, which can be used as reference values.

Although both ALP and BAP are commonly used bone formation markers, we selected ALP over BAP because ALP is still the most widely used routine laboratory test.^24^ Admittedly, ALP consists of several isoenzymes including liver, bone, placenta, and intestine, therefore total ALP does not specifically represent bone formation. Yet, assessing BAP does not offer a distinct advantage in infants, as the liver isozyme’s contribution to total ALP activity is minimal in children experiencing increased bone turnover.^3^ Notably, ALP3, the bone type, was the dominant isoenzyme in all measurable cases in this study, validating the use of total ALP as a bone absorption marker during early infancy. Our results demonstrated that ALP and iPTH levels were elevated in response to vitamin D deficiency with a biphasic or negative linear pattern detected by the spline curve regression analysis. These results are all in line with the previous reports, demonstrating the reproducibility and legitimacy of our analysis.

Although ALP levels are commonly associated with rickets and osteomalacia associated with hypophosphatemia, this study primarily focused on infants during routine well-baby checkups. Furthermore, all infants in this study had serum phosphorus levels above the lower limit of the monthly range, and none displayed signs of growth delay. Thus, the observed elevation in ALP levels among these infants likely indicates subclinical vitamin D deficiency.

TRACP-5b and NTx, 2 bone absorption markers measured in this study, have also been poorly investigated during early infancy. Our TRACP-5b measurements for the early neonatal period and the previously reported data for full-term infants were almost identical.^18^ Since the possible influence of ABO blood type upon TRACP-5b values has been previously documented,^25^ we additionally performed a sub-analysis for blood type in both vitamin D deficient and sufficient groups, confirming no differences found in either group (data not shown). Lapillonne et al. presented urine NTx reference values for 70 healthy infants aged 0 to 375 days.^26^ Briana et al. examined cord blood and day 1 and day 4 serum NTx levels in appropriate-for-gestational-age (AGA) and SGA infants separately.^27^ Only 6 SGA infants (1.3%) were included out of 456 eligible participants in our study, while neonatal bone turnover in SGA cases may differ from bone metabolism in AGA subjects.^28^ Nevertheless, no previous studies have investigated both TRACP-5b and NTx simultaneously in the context of vitamin D deficiency during early infancy, making our studies significant.

Initially, we anticipated that both serum TRACP-5b and NTx levels would be elevated in the vitamin D-deficient group, assuming that accelerated bone metabolic turnover in postnatal vitamin D deficiency would stimulate both bone formation and resorption in a hypermetabolic turnover-type bone loss state.^29^ However, neither TRACP-5b nor NTx showed significant differences between the vitamin D deficiency and the sufficiency groups. Furthermore, no characteristic dose–response correlations were manifested with the spline curve regression analysis. These results are inconsistent with previous studies showing elevated serum TRACP-5b and NTx levels in response to clinical vitamin D deficiency during late infancy or teenage.^6^^,^^11^

The reasons why TRACP-5b and NTx did not increase in response to vitamin D deficiency among our study subjects can only be speculated. TRACP-5b and NTx may not be as sensitive markers as ALP, especially in the case of subclinical vitamin D deficiency like in our study. It is also possible that sufficient oral intake of calcium from breast milk or formula, as indicated by the absence of hypocalcemic subjects in the laboratory data, may have contributed to the results. It is important to note that bone metabolism in infants is different from that in adults, as it requires both growth and bone remodeling. Interpretation of bone markers is more complicated in infants as they reflect the cumulative effect of growth and remodeling processes, and bone resorption may be relatively suppressed during infancy when vigorous bone growth is prioritized.

The absence of appropriate reference values in early infancy has hindered the utilization of BTMs other than ALP in clinical practice. Fischer et al.^30^ reported a TRACP-5b reference range of 0.1–21 years of age and Rauchenzauner et al.^31^ reported 2 months to 18 years, despite their small sample numbers with 9 and 11 patients who were under age 1 yr, respectively, and could not be examined by age in months. However, our study has established reference ranges for TRACP-5b and NTx in postnatal infants. Lapillonne et al. reported urine NTx/Cr values in infancy; urine NTx/Cr increased shortly after birth, peaking at 36 days of age and then declining until about 4 months of age.^26^ In contrast, our results showed that serum NTx values were relatively high up to 1 month of age and gradually decreased thereafter. The observed discrepancy in the trend between urine and serum NTx levels might be attributed to changes in the physiological glomerular filtration rate (GFR) shortly after birth,^32^ as suggested by previous studies indicating a correlation between serum NTx levels and renal function.^4^

This study has several limitations. First, due to the nature of this single-center study, the generalizability of our findings to all infants may be limited. Furthermore, the optimal sample size needed to detect differences was not precalculated but was determined in an exploratory manner, and the potential for selection bias exists as participants had a clinical indication for taking a blood sample. Second, only ALP was examined as a marker of bone formation. The inclusion of other bone formation markers along with ALP levels could have provided a more reliable assessment of bone formation. Third, only serum Cr levels were reported, and GFR was not measured in this study. Although estimating GFR from serum Cr levels alone is challenging especially in infants, we assumed that there was no difference in serum NTx levels between the 2 groups by showing that serum Cr levels in both vitamin D-sufficient and insufficient groups were within the monthly age reference values and that no difference was observed between the 2 groups. Fourth, the limited relevance of this study to clinical practice should be acknowledged, as the resorption markers reported here are not necessarily used in routine clinical care worldwide. Lastly, BMD was not evaluated due to the ethical concerns related to radiation.

In summary, we retrospectively investigated the impact of subclinical vitamin D deficiency on BTMs in Japanese infants. Bone formation markers like ALP were significantly elevated in response to vitamin D deficiency, yet no significant changes in bone resorption markers including TRACP-5b and NTx were found. Additionally, our study presents the first postnatal reference values of serum TRACP-5b and NTx, revealing the unique course of serum NTx compared to urine NTx. Overall, these data provide novel insights into the complex interplay between born formation and absorption during infancy and the impact of vitamin D deficiency on this process.

**Highlights**

- ALP, a marker of bone formation, and iPTH are elevated in vitamin D deficiency in early infants, whereas TRACP-5b and NTx, the bone resorption markers, were not affected by vitamin D deficiency in this population.
- TRACP-5b levels were low in the early neonatal period but increased in the first month of life and remained relatively high thereafter. Serum NTx levels, on the other hand, were relatively high up to 1 mo of age and gradually decreased thereafter.

## Data Availability

The datasets used and/or analyzed during the current study are available from the corresponding author upon reasonable request.
